# Literary Notes

**Published:** 1907-09

**Authors:** 


### LITERARY NOTES.

---

Dr. Herbert L. Burrell, president-elect of the American Medical Association, is now preparing the manuscript of a work on surgery, of considerable magnitude and importance, which the Philadelphia publishers, P. Blakiston's Son & Co., will bring out. This is another indication of Dr. Burrell's tireless labor in educational fields. The book is to be complete in one royal octavo volume, well illustrated, and, needless to add, well and authoritatively written.

---

The Cleveland Press, of Chicago, announces the early issue of a biography of Nathan Smith Davis, sometimes called the "father of the American Medical Association." Dr. Isaac N. Danforth, of Chicago, is preparing this work, which will possess great interest to the admirers of Dr. Davis.

